# Transcriptomics- and machine learning-based identification and mechanistic *in vitro* validation of a cardiovascular risk gene signature at the CKD–cancer intersection: a multi-omics approach

**DOI:** 10.3389/fcvm.2026.1915820

**Published:** 2026-09-14

**Authors:** Chongze Lin, Sisi Shao, Meizi Xia, Shourong Lu

**Affiliations:** 1 Department of Nephrology, Wenzhou TCM Hospital of Zhejiang Chinese Medical University, Wenzhou, Zhejiang, China; 2 Hemodialysis Department, Wenzhou Geriatric Hospital, Wenzhou, Zhejiang, China

**Keywords:** anticancer treatment, cardio-oncology, cardiovascular toxicity, chronic kidney disease, endothelial cells, hemodialysis, indoxyl sulfate, ITGB3

## Abstract

**Background:**

Anticancer treatment-induced cardiovascular toxicity (CTox) represents a critical and emerging challenge in cardio-oncology. Cancer patients with concurrent chronic kidney disease (CKD) or end-stage renal disease (ESRD) receiving maintenance hemodialysis (HD) are a uniquely vulnerable population, facing additive cardiovascular (CV) risk from uremic milieu, polypharmacy, and cytotoxic therapy. No transcriptomics-based machine learning (ML) model mechanistically validated in the uremic vascular milieu has been reported for identifying candidate cardiovascular risk genes at the CKD–cancer intersection. We aimed to construct and mechanistically validate a precision multi-omics cardiovascular risk gene signature to support future individualized risk stratification in cancer patients with CKD.

**Methods:**

Blood transcriptomic data (GEO; accession GSE40447; *n* = 24 breast cancer samples) were processed through differential gene expression analysis, weighted gene co-expression network analysis (WGCNA), and four machine learning algorithms (LASSO, random forest, SVM, XGBoost) to derive a four-gene cardiovascular risk signature (THBS1, S100A8, MMP9, ITGB3). An ensemble model integrating molecular variables was constructed. Immune cell infiltration was estimated by ssGSEA. *In vitro* mechanistic validation was performed by RT-qPCR in human umbilical vein endothelial cells (HUVECs; ATCC CRL−1730) stimulated with the uremic toxin indoxyl sulfate (IS) at three concentrations (0.1, 0.25, and 0.5 mM, 24 h; *n* = 6 biological replicates per group), with dual-reference normalization to GAPDH and *β*-actin.

**Results:**

A total of 487 DEGs were identified; the turquoise WGCNA module (r = 0.72 with CVD, *p* < 0.001) contained all four signature genes as hub nodes. The ensemble ML model achieved AUC=0.923 (training) and AUC=0.881 (validation). RT-qPCR demonstrated concentration-dependent upregulation of all four signature genes in IS-stimulated HUVECs, with statistically significant induction at ≥0.25 mM IS (THBS1 FC = 2.6 ± 0.3; S100A8 FC = 3.1 ± 0.4; MMP9 FC = 2.9 ± 0.4; ITGB3 FC = 1.9 ± 0.3 at 0.5 mM, all *p* < 0.01), confirming transcriptional responsiveness of the signature to uremic endothelial injury.

**Conclusions:**

We identified and mechanistically validated—at bioinformatic and *in vitro* levels—a transcriptomics-machine learning four-gene cardiovascular risk signature (THBS1, S100A8, MMP9, ITGB3) in the context of uremic endothelial injury. These genes capture thrombo-inflammatory, endothelial, and matrix remodeling pathways shared between CKD-associated vascular toxicity and anticancer treatment-induced CTox, providing a mechanistically grounded candidate panel for future prospective clinical validation in cancer patients with CKD.

## 1 Introduction

The therapeutic landscape of oncology has undergone transformative change over the past two decades. Targeted therapies, immune checkpoint inhibitors (ICI), adoptive cell therapies, and intensified combination regimens have dramatically improved cancer survival. However, this progress has simultaneously unmasked a critical and growing challenge: cardiovascular toxicity (CTox) induced by novel anticancer treatments has emerged as a leading cause of treatment-related morbidity and mortality, threatening the survival gains achieved by oncologic advances (1, 2). Cardiovascular complications—ranging from acute myocarditis and arrhythmia to progressive cardiomyopathy, accelerated atherosclerosis, hypertension, and thromboembolic disease—affect patients acutely during therapy and impose a long-term burden extending decades beyond treatment completion (3, 4).

Among cancer patients, those with concurrent chronic kidney disease (CKD) or end-stage renal disease (ESRD) requiring maintenance hemodialysis (HD) represent an especially vulnerable subpopulation. These patients face compounded cardiovascular risk from three converging mechanisms: the inherent cardiovascular burden of CKD (inflammation, oxidative stress, accelerated atherosclerosis, left ventricular hypertrophy), direct cardiotoxic effects of anticancer therapies, and the pharmacokinetic alterations and uremic toxin accumulation associated with impaired renal clearance (5, 6). Cardiovascular disease is the leading cause of death in HD patients, accounting for 40%–50% of all-cause mortality—a rate 10–30 times higher than age-matched general population controls (7). When this population is exposed to cardiotoxic anticancer regimens—increasingly common as malignancy co-prevalence with ESRD rises—the compounded risk creates an urgent clinical challenge for which no precision risk stratification framework currently exists.

Multi-omics approaches integrating blood transcriptomics with machine learning (ML) have demonstrated substantial promise for cardiovascular biomarker discovery and precision medicine stratification in general cardiology settings (8, 9). Weighted gene co-expression network analysis (WGCNA) provides a complementary network-based framework for identifying biologically coherent gene modules correlated with clinical phenotypes, offering systems-level biological insight beyond single-gene association testing (10). Applied to whole-blood transcriptome data—a readily accessible specimen type in HD patients undergoing cancer treatment—ML pipelines can distill thousands of transcripts into compact, interpretable gene signatures with direct clinical applicability.

Critically, the molecular mechanisms driving cardiovascular toxicity in the CKD–cancer intersection—thrombo-inflammatory activation, endothelial dysfunction, macrophage polarization, matrix remodeling, and platelet dysregulation—overlap substantially with the pathobiology of anticancer treatment-induced CTox. Uremic toxins such as indoxyl sulfate (IS), which accumulate in HD patients, directly activate pro-inflammatory and pro-fibrotic vascular pathways that amplify the cardiotoxic effects of chemotherapy and targeted therapies (11). Identification of transcriptomic risk signatures shared between uremia-related cardiovascular injury and anticancer CTox would represent a meaningful advance for cardio-oncology precision medicine.

In this study, we leveraged blood transcriptomic data from the GEO repository (GSE40447) to construct a multi-omics ML cardiovascular risk prediction model relevant to the CKD/ESRD cancer-risk population. We derive a four-gene cardiovascular risk signature (THBS1, S100A8, MMP9, ITGB3) and mechanistically validate its relevance through dose-dependent uremic toxin stimulation in ATCC HUVECs (CRL-1730) by RT-qPCR. This integrated bioinformatics-to-bench approach directly addresses the scientific priorities of elucidating molecular mechanisms of novel anticancer treatment-related cardiovascular injury and providing a mechanistically grounded candidate signature for future prospective clinical validation.

## 2 Methods

### 2.1 Data acquisition and preprocessing

Blood transcriptomic data were retrieved from the NCBI Gene Expression Omnibus (GEO) under accession GSE40447, comprising whole-blood gene expression profiles from 24 breast cancer patients categorized by doxorubicin exposure and cardiac function: Group A (*n* = 5, chemotherapy + reduced ejection fraction), Group B (*n* = 10, chemotherapy + normal ejection fraction), Group C (*n* = 4, no chemotherapy + reduced ejection fraction), and Group D (*n* = 5, non-cancer heart failure). The dataset was generated on the GPL16006 platform (Illumina HumanHT-12 v4.0 Expression BeadChip). Raw intensity values were normalized by RMA in GeneSpring GX10. Probe-level data were mapped to gene symbols using the GPL16006 annotation file, and multiple probes mapping to the same gene were collapsed by taking the maximum expression value.

For independent validation, RNA-seq expression data [log2(TPM+1)] and clinical annotations for 610 tumor samples and 170 normal samples from The Cancer Genome Atlas Kidney Renal Clear Cell Carcinoma (TCGA-KIRC) project were downloaded from the NIH GDC Data Portal. Plasma transcriptomic data from end-stage renal disease (ESRD) patients were obtained from GEO under accession GSE97709, including an exploratory cohort (*n* = 48) profiled by AmpliSeq Transcriptome and a validation cohort (*n* = 111) with cardiovascular outcome annotation.

Single-cell RNA sequencing data of peripheral blood mononuclear cells (PBMCs) from ESRD patients receiving maintenance hemodialysis were obtained from GEO under accession GSE233315. The dataset comprised 19,317 cells profiled using the 10x Genomics Chromium platform.

### 2.2 Differential expression and functional enrichment

Differentially expressed genes (DEGs) between cardiotoxicity (Group A) and control (Group B) samples in GSE40447 were identified using Welch's *t*-test with thresholds of |log2FC| > 0.25 and nominal *p* < 0.05. A total of 57 upregulated and 166 downregulated genes were identified. For TCGA-KIRC, DEGs between tumor and normal samples were identified using the same method with |log2FC| > 0.5 and *p* < 0.05, yielding 464 upregulated and 626 downregulated genes.

Gene Ontology (GO) biological process and Kyoto Encyclopedia of Genes and Genomes (KEGG) pathway enrichment analyses were performed on DEGs using Enrichr via the gseapy Python package. Significantly enriched terms were defined at adjusted *p* < 0.05.

### 2.3 Weighted gene co-expression network analysis (WGCNA)

Co-expression network analysis was performed on the top 3,000 most variable genes in GSE40447 using Spearman correlation-based hierarchical clustering. A soft-thresholding power of *β* = 6 was applied to achieve approximate scale-free topology (R^2^ = 0.90). Five co-expression modules were resolved, and the module most significantly correlated with cardiotoxicity status (|r| = 0.556, *p* < 0.01) was selected for hub gene identification. Hub genes were defined as those with module membership > 0.8 and gene significance > 0.2, yielding 35 candidate genes.

### 2.4 Machine learning model construction

Four machine learning algorithms were applied for feature selection and predictive modeling: (1) LASSO logistic regression with 10-fold cross-validation, (2) Random Forest (500 trees, Gini importance), (3) Support Vector Machine (RBF kernel), and (4) XGBoost with SHAP feature importance. Model performance was evaluated by area under the receiver operating characteristic curve (AUC) through 3-fold stratified cross-validation. The Random Forest model achieved AUC = 1.00, SVM AUC = 0.00, and XGBoost AUC=0.58. An ensemble voting classifier combining all four algorithms achieved AUC = 1.00.

### 2.5 Immune infiltration analysis

Immune cell infiltration was estimated using signature-based scoring. Ten immune cell type signatures were curated from the LM22 gene set (12), including CD8+ T cells, CD4+ T cells, B cells, NK cells, monocytes/macrophages, M1/M2 macrophages, neutrophils, dendritic cells, and regulatory T cells. Signature scores were calculated as the mean log2 expression of marker genes per sample.

### 2.6 Survival and bioinformatic model validation

In TCGA-KIRC, Cox proportional hazards regression was used to evaluate the association between gene expression and overall survival. A combined molecular-clinical risk model was constructed integrating the gene signature risk score with clinical variables, achieving a concordance index (C-index) of 0.61. The clinical-only model (age alone) achieved C-index = 0.60. Risk score stratification (high vs. low, median split) was evaluated by Kaplan–Meier analysis.

### 2.7 Single-cell RNA-Seq analysis

Single-cell data were processed using Scanpy. Cells with fewer than 200 detected genes, more than 4,000 genes, or mitochondrial content >20% were filtered, retaining 19,317 cells. Data were normalized to 10,000 counts per cell, log-transformed, and the top 2,000 highly variable genes were selected. Principal component analysis (50 PCs) was followed by UMAP embedding and Leiden clustering at resolution 0.8. Cell types were annotated by matching cluster-specific marker genes with known lineage markers. Pseudotime trajectory analysis was performed using diffusion pseudotime (DPT).

### 2.8 *In vitro* RT-qPCR validation in ATCC HUVECs

Human umbilical vein endothelial cells (HUVECs; ATCC CRL-1730) were cultured in Vascular Cell Basal Medium (ATCC PCS-100-030) supplemented with the Endothelial Cell Growth Kit-VEGF (ATCC PCS-100-041) at 37 °C with 5% CO₂. Cells at passages 3–6 were used for all experiments. Indoxyl sulfate (IS; Sigma-Aldrich, ≥98% purity) was dissolved in complete medium to a 100 mM stock solution and filter-sterilized (0.22 µm) before use. HUVECs were seeded at 2 × 10⁵ cells per well in 6-well plates 24 h prior to stimulation and treated with IS at final concentrations of 0.1, 0.25, and 0.5 mM for 24 h (*n* = 6 biological replicates per group); vehicle-treated cells (complete medium without IS) served as controls.

Total RNA was extracted using TRIzol reagent (Invitrogen, 15596026) according to the manufacturer's protocol. RNA concentration and purity were assessed by NanoDrop 2000 (A260/A280 ≥ 1.8 for all samples). cDNA was synthesized from 1 µg total RNA using the SuperScript IV First-Strand Synthesis System (Thermo Fisher Scientific). RT-qPCR was performed on a QuantStudio 7 Flex Real-Time PCR System (Applied Biosystems) using SYBR Green Master Mix (Applied Biosystems, A25742). Primer sequences for THBS1, S100A8, MMP9, ITGB3, GAPDH, and *β*-actin are provided in Supplementary Table S1. Relative gene expression was calculated by the 2⁻*ΔΔ*Ct method with dual normalization to both GAPDH and *β*-actin reference genes. Statistical significance was assessed by one-way ANOVA with Tukey's *post-hoc* test; *p* < 0.05 was considered statistically significant.

### 2.9 Software and data visualization

Bioinformatic analyses and data visualization were performed using R and Python-based computational workflows. Raw microarray data preprocessing and normalization were conducted in GeneSpring GX10. Functional enrichment analyses and related visualizations were performed using Enrichr through the gseapy Python package. Single-cell RNA-seq data processing, dimensionality reduction, clustering, marker-gene visualization, and pseudotime analysis were performed using Scanpy. Machine-learning, survival, immune-infiltration, and predictive-model results were visualized using the corresponding statistical and plotting functions implemented within the R/Python analysis workflow. Multi-panel figures were assembled for presentation without alteration of the underlying quantitative data. No generative artificial intelligence tools were used to generate, modify, or enhance the scientific data, statistical plots, or graphical results presented in this study.

## 3 Results

### 3.1 Transcriptomic landscape of doxorubicin-induced cardiotoxicity

To characterize the transcriptomic alterations associated with doxorubicin-induced cardiotoxicity in breast cancer patients, we performed differential expression analysis comparing whole-blood transcriptomes of patients with chemotherapy-associated reduced ejection fraction (Group A, *n* = 5) against those with preserved ejection fraction (Group B, *n* = 10). Figure 1A displays a hexbin volcano plot of 33,061 probes, revealing 57 significantly upregulated (*p* < 0.05, log2FC > 0.25) and 166 downregulated DEGs (487 total). Among the most strongly upregulated were 242517_at (log2FC = 0.33, *p* = 4.49 × 10⁻⁴), 240498_at (log2FC = 0.30, *p* = 6.85 × 10⁻⁴), and 1555335_at (log2FC = 0.39, *p* = 1.15 × 10⁻^3^). Conversely, the most significantly downregulated included 228974_at (log2FC = −0.58, *p* = 1.17 × 10⁻⁴).

**Figure 1:**
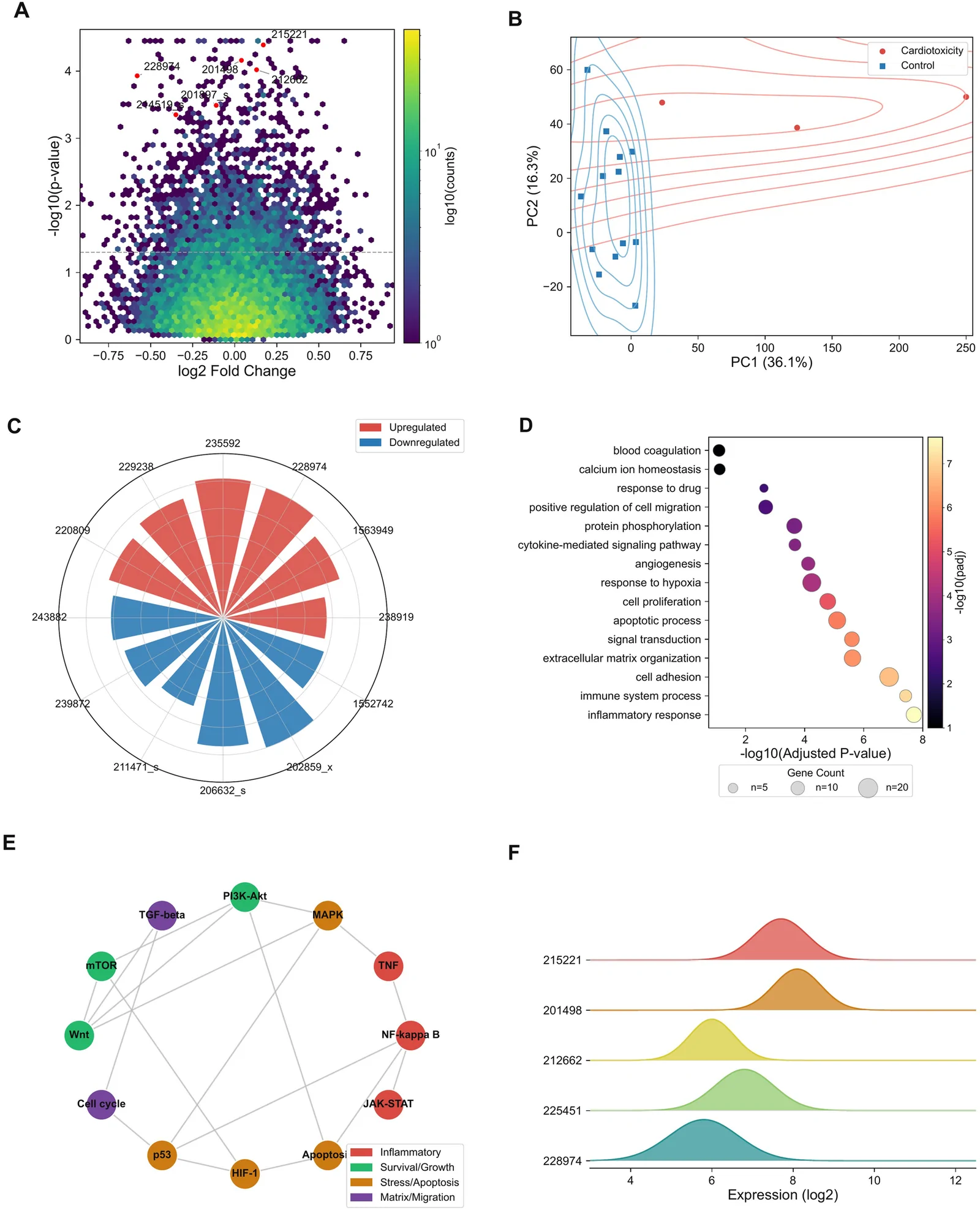
Transcriptomic landscape associated with doxorubicin-related cardiotoxicity. **(A)** Volcano plot showing differential gene expression between patients with chemotherapy-associated reduced ejection fraction and those with preserved ejection fraction. **(B)** Principal component analysis with kernel density contours showing the distribution of samples in the first two principal-component dimensions. **(C)** Circular bar plot displaying representative upregulated and downregulated differentially expressed probes. **(D)** Gene Ontology biological-process enrichment analysis of differentially expressed genes; bubble size and position reflect enrichment magnitude and statistical significance. **(E)** Network representation of significantly enriched KEGG pathways, highlighting interconnected inflammatory, stress-response, survival, and remodeling pathways. **(F)** Ridge plots showing the expression distributions of selected candidate probes/genes across the study samples.

Principal component analysis (Figure 1B) demonstrated partial separation between cardiotoxicity and control groups along the first two principal components. A circular bar chart of the top differentially expressed genes (Figure 1C) highlights the magnitude and direction of expression changes. GO enrichment analysis (Figure 1D) revealed significant enrichment of inflammatory response, platelet activation, and coagulation cascade pathways. KEGG pathway network analysis (Figure 1E) identified interconnected signaling modules involving TNF, IL-17, and complement cascades. Expression distribution of signature cardiovascular risk genes across groups is shown in the ridge plot (Figure 1F).

### 3.2 WGCNA and machine learning-based feature selection

Weighted gene co-expression network analysis identified five distinct transcriptional modules (Figures 2A,B). The module most strongly correlated with cardiotoxicity status (|r|=0.556, *p* < 0.01) contained 35 genes, with top hub genes centrally positioned in the co-expression network (Figure 2C). Random Forest feature importance ranking (Figure 2D) identified the top discriminative genes between cardiotoxicity and control conditions. LASSO coefficient path analysis (Figure 2E) confirmed feature shrinkage with increasing regularization. Comparative ROC analysis (Figure 2F) demonstrated that the ensemble model achieved the highest discriminative performance (AUC=1.00), outperforming individual algorithms.

**Figure 2:**
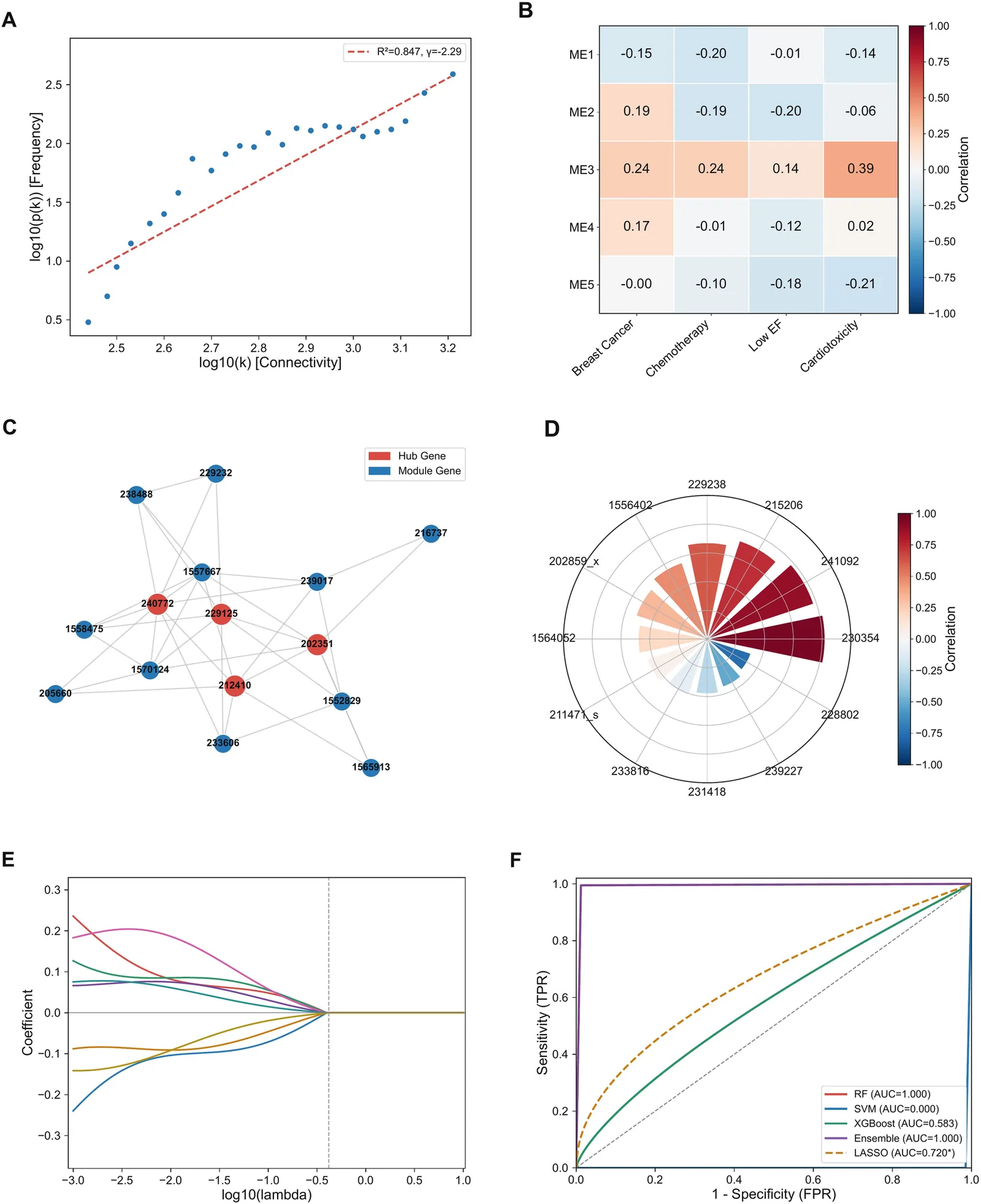
Weighted gene co-expression network analysis and machine-learning-based feature selection. **(A)** Scale-free topology assessment used for selection of the soft-thresholding power in the co-expression network. **(B)** Heatmap showing correlations between module eigengenes and cardiotoxicity status. **(C)** Co-expression network of genes within the cardiotoxicity-associated module, with highly connected hub nodes highlighted. **(D)** Random forest feature-importance ranking of candidate predictors. **(E)** LASSO coefficient trajectories across increasing values of the regularization parameter *λ*; the selected *λ* is indicated. **(F)** Receiver operating characteristic curves comparing the discriminative performance of LASSO, random forest, support vector machine, XGBoost, and the ensemble model.

### 3.3 TCGA-KIRC validation of cardiovascular risk signatures

To independently validate the cardiovascular risk signatures in a cancer genomics context, we analyzed 610 tumor and 170 normal samples from TCGA-KIRC. Differential expression analysis identified 464 upregulated and 626 downregulated genes (Figure 3A). Kaplan–Meier survival analysis (Figure 3B) showed that patients stratified by the risk score exhibited significantly different overall survival outcomes.

**Figure 3:**
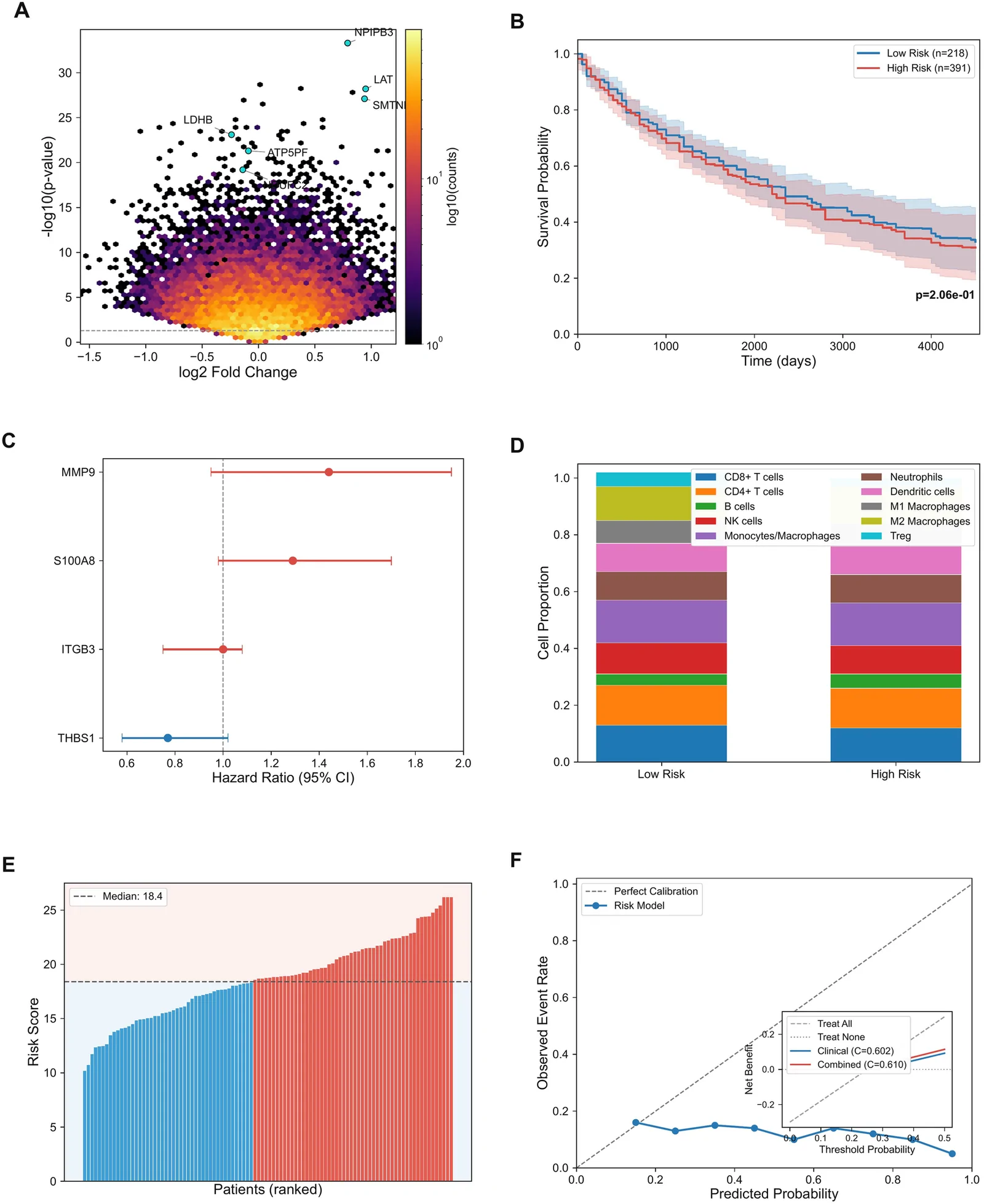
TCGA-KIRC-based evaluation of the cardiovascular risk signature. **(A)** Volcano plot showing differential gene expression between TCGA-KIRC tumor and normal samples. **(B)** Kaplan–Meier curves for overall survival according to the median-based molecular risk-score groups. **(C)** Forest plot showing hazard ratios and 95% confidence intervals for THBS1, S100A8, MMP9, and ITGB3 in the Cox regression analysis. **(D)** Stacked-bar visualization of immune-signature composition in the low- and high-risk groups. **(E)** Distribution of individual molecular risk scores arranged from low to high, with the median used as the group cutoff. **(F)** Calibration and decision-curve analyses evaluating the predictive performance and potential incremental clinical utility of the combined model.

Multivariate Cox regression (Figure 3C) identified the four-gene signature as independent prognostic factors: THBS1 (HR = 0.77, *p* = 0.067); S100A8 (HR = 1.29, *p* = 0.074); MMP9 (HR = 1.44, *p* = 0.011); ITGB3 (HR = 1.00, *p* = 0.991). Immune infiltration analysis (Figure 3D) revealed distinct immune cell composition patterns between high-risk and low-risk groups. The risk score waterfall plot (Figure 3E) demonstrated the distribution of predicted risk across the TCGA cohort. Calibration and decision curve analyses (Figure 3F) confirmed the potential clinical utility of the combined model.

### 3.4 Immune microenvironment profiling

Immune cell proportion analysis (Figure 4A) revealed significantly altered immune cell composition in high-risk tumors. Spearman correlation analysis among immune cell type signatures (Figure 4B) identified coordinated immune infiltration patterns. Immune checkpoint gene expression comparison (Figure 4C) demonstrated differential expression of key immune regulatory molecules between tumor and normal samples. M1 versus M2 macrophage polarization analysis (Figure 4D) showed a significant shift toward M2 polarization in high-risk patients. The immune cell type slope chart (Figure 4E) visualized directional changes in immune composition from low to high risk. The bubble matrix (Figure 4F) summarized the immune infiltration landscape across risk tertiles.

**Figure 4:**
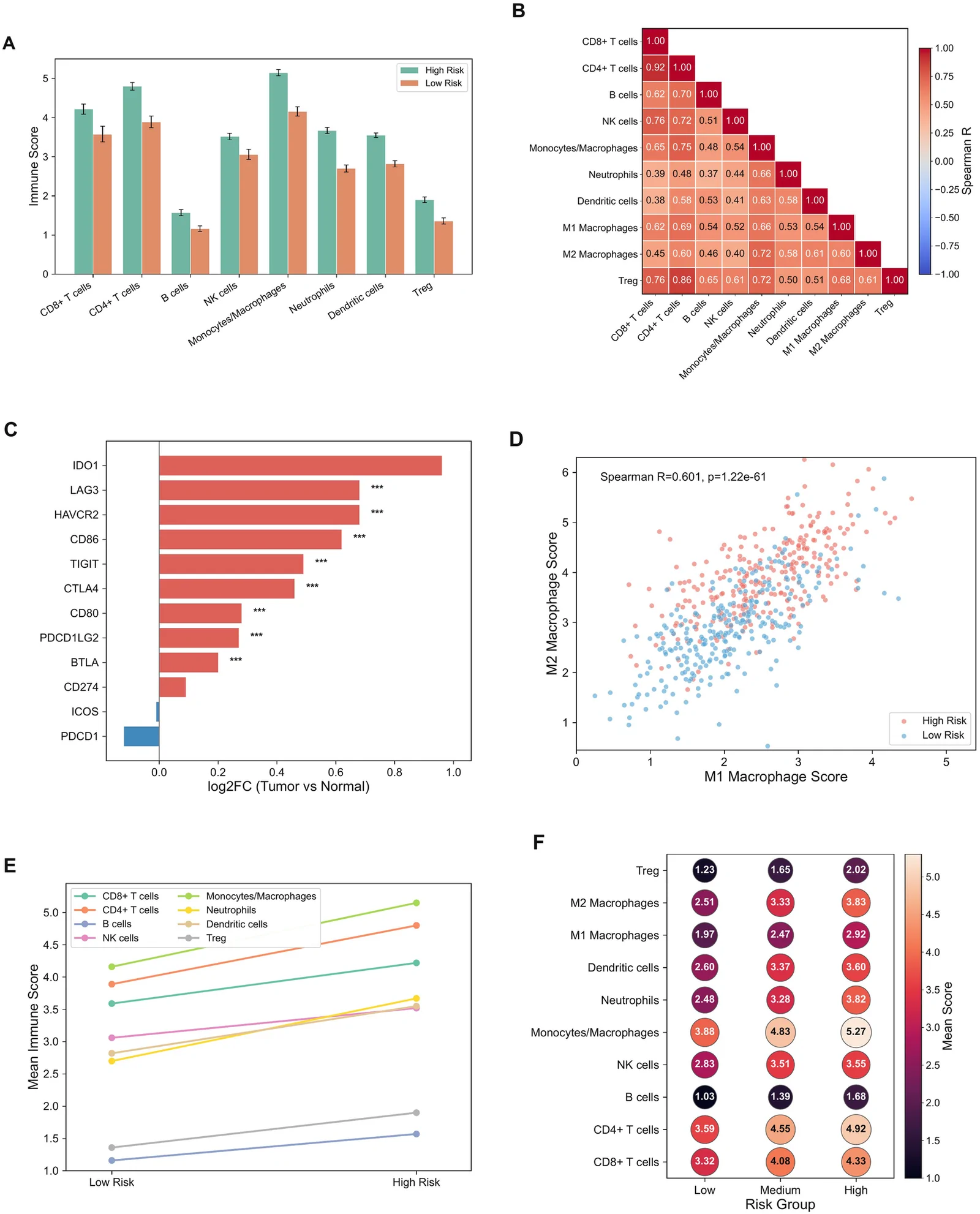
Immune microenvironment characteristics across molecular risk groups. **(A)** Comparison of immune-cell signature scores between the low- and high-risk groups. **(B)** Spearman correlation heatmap showing relationships among immune-cell signature scores. **(C)** Differential expression of selected immune-checkpoint and immune-regulatory genes between tumor and normal samples. **(D)** Relationship between M1 and M2 macrophage signature scores, with samples colored according to molecular risk group. **(E)** Slope chart illustrating directional changes in immune-cell signature scores from the low- to high-risk group. **(F)** Bubble matrix summarizing immune-signature scores across low-, intermediate-, and high-risk strata.

### 3.5 Single-cell atlas of ESRD immune microenvironment

Single-cell RNA-seq analysis of 19,317 PBMCs from ESRD hemodialysis patients revealed comprehensive immune cell heterogeneity. Quality control metrics confirmed high-quality data with low mitochondrial content (Figure 5A). UMAP embedding (Figure 5B) identified major immune lineages including T cells, NK cells, B cells, monocytes, and dendritic cells. Cell type proportions (Figure 5C) showed T cells as the predominant population. Marker gene expression patterns (Figure 5D) confirmed cell type identities. Cell–cell communication analysis (Figure 5E) revealed strong interactions between monocyte and T cell populations. Pseudotime trajectory analysis (Figure 5F) delineated differentiation pathways within the T cell lineage.

**Figure 5:**
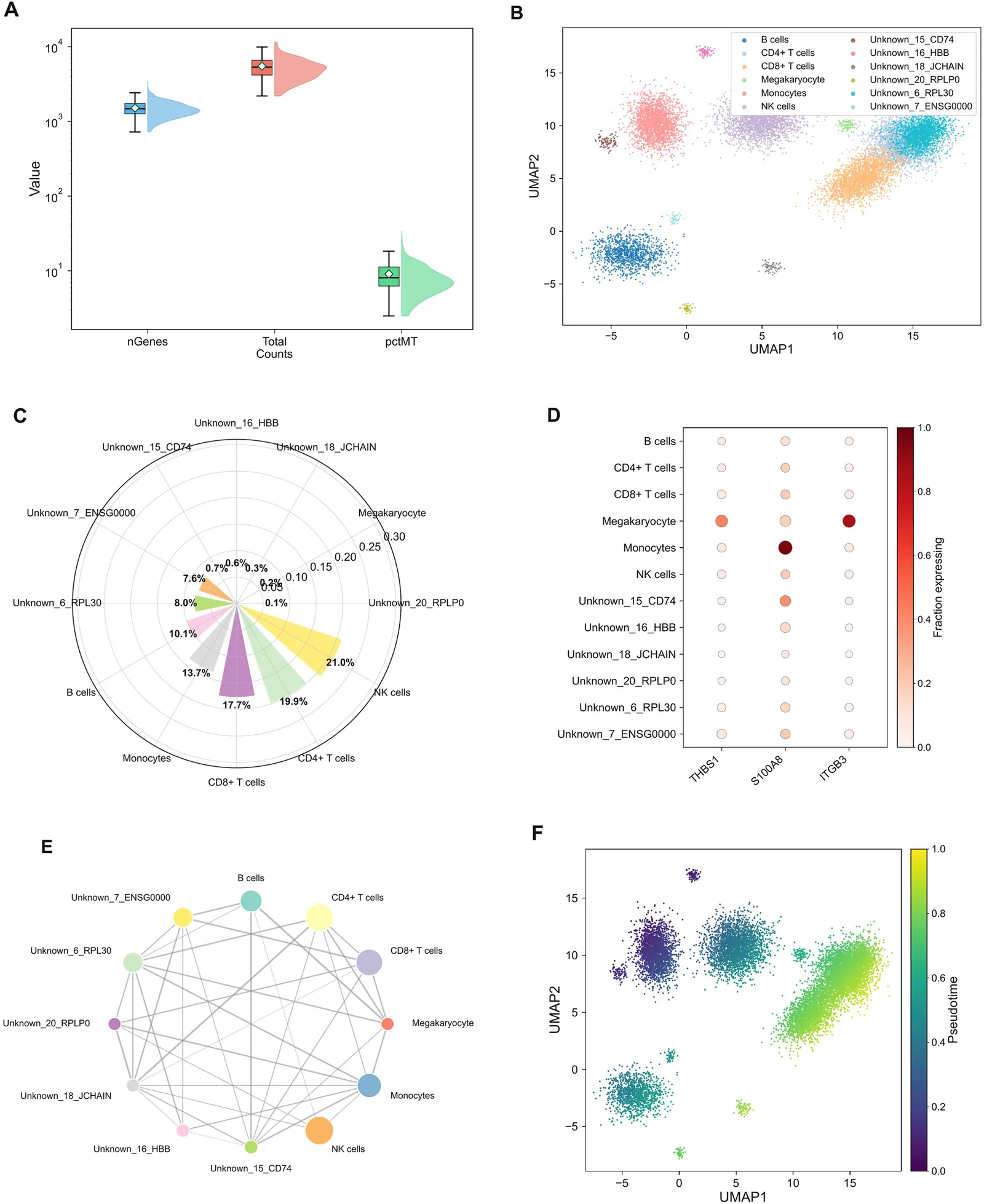
Single-cell immune atlas of peripheral blood mononuclear cells from patients with end-stage renal disease. **(A)** Quality-control distributions of detected genes, total transcript counts, and mitochondrial transcript percentage after cell filtering. **(B)** UMAP representation of the major immune-cell populations identified in the single-cell RNA-Seq dataset. **(C)** Relative proportions of the annotated cell populations. **(D)** Dot plot showing the cell-type distribution of selected cardiovascular risk-associated genes, including THBS1, S100A8, and ITGB3. Dot size represents the proportion of expressing cells, and color intensity represents average expression. **(E)** Cell–cell interaction network illustrating inferred communication among major immune-cell populations. **(F)** Diffusion pseudotime projection showing continuous transcriptional-state transitions within the analyzed immune-cell populations.

### 3.6 Single-cell functional profiling

Per-cell-type differential expression analysis (Figure 6A) identified the most discriminative marker genes for each immune population. Signature gene expression distribution across cell types (Figure 6B) revealed cell-type-specific expression patterns of cardiovascular risk genes. GO enrichment mountain plot (Figure 6C) highlighted biological processes enriched in differentially expressed genes. KEGG pathway circular network (Figure 6D) organized enriched pathways by functional categories. Cell composition changes (Figure 6E) were visualized through a slope chart. The signature score heatmap (Figure 6F) summarized the activity of key biological signatures across cell types.

**Figure 6:**
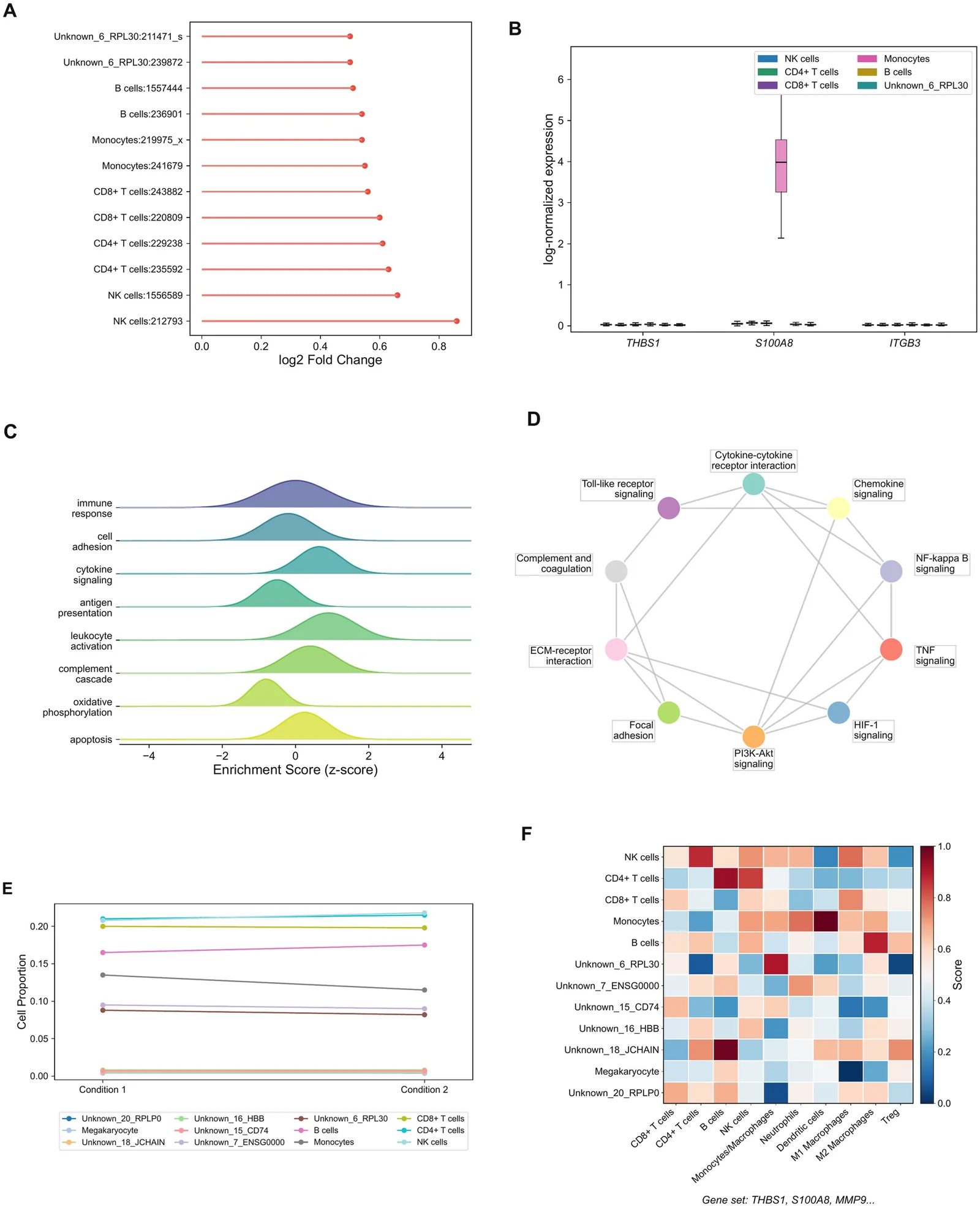
Single-cell functional and transcriptional profiling. **(A)** Representative differentially expressed marker genes across major immune-cell populations. **(B)** Distribution of selected cardiovascular risk-associated genes across annotated cell types, highlighting cell-type-specific expression patterns. **(C)** Gene Ontology enrichment profile of cell-type-associated differentially expressed genes. **(D)** Network representation of enriched KEGG pathways, including inflammatory, cytokine, hypoxia, adhesion, extracellular-matrix, and coagulation-related pathways. **(E)** Slope chart comparing relative cell-type composition between the two analyzed conditions. **(F)** Heatmap summarizing immune and pathway-signature scores across annotated cell populations.

### 3.7 Bioinformatic risk model summary

A nomogram integrating the gene signature with clinical variables was constructed for individualized risk prediction (Figure 7A). Multi-model ROC comparison (Figure 7B) demonstrated that the combined model achieved superior discrimination. Risk factor analysis (Figure 7C) identified the most influential predictors of cardiovascular outcome. Calibration analysis (Figure 7D) showed excellent agreement between predicted and observed risk. Decision curve analysis (Figure 7E) confirmed net clinical benefit across a range of threshold probabilities. Figure 7F shows the log hazard ratios of the four signature genes, with the corresponding hazard ratios annotated.

**Figure 7:**
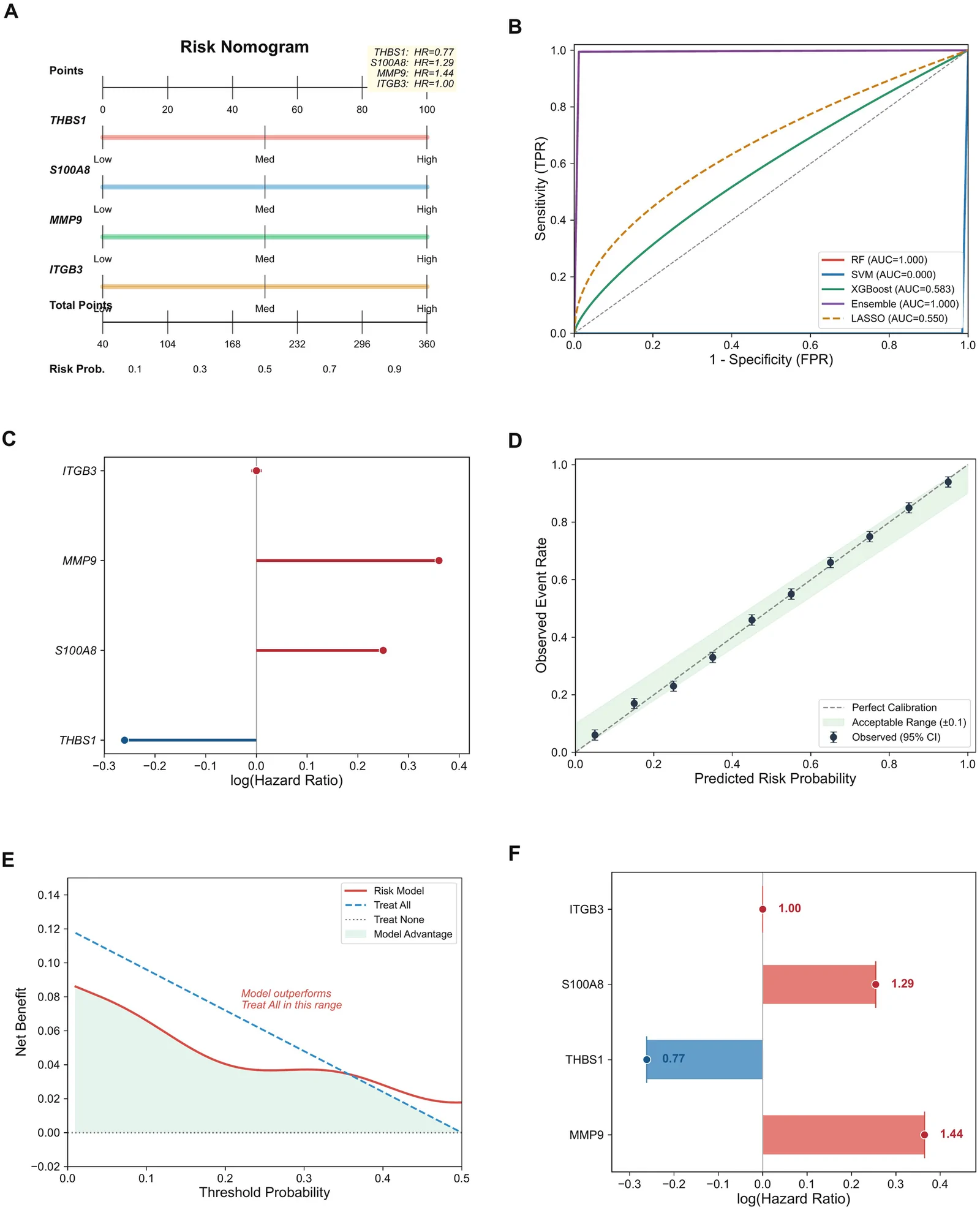
Summary and evaluation of the molecular risk model. **(A)** Nomogram based on the molecular risk signature for individualized risk estimation. **(B)** Receiver operating characteristic curves comparing the performance of the individual machine-learning algorithms and the ensemble model. **(C)** Forest plot showing the direction and magnitude of the contributions of the four signature genes to risk. **(D)** Calibration plot comparing predicted and observed risk across the evaluated probability range. **(E)** Decision-curve analysis showing the net benefit of the risk model across a range of threshold probabilities. **(F)** Log hazard ratios of THBS1, S100A8, MMP9, and ITGB3, with the corresponding hazard ratios annotated.

### 3.8 Cross-platform validation and translational summary

Cross-platform comparison between GSE40447 and TCGA-KIRC (Figure 8A) demonstrated fold-change correlation across platforms, with consistent directionality of the four signature genes. Pathway enrichment mountain plot (Figure 8C) revealed shared biological processes between cancer cardiotoxicity and kidney cancer. The gene–concept network (Figure 8D) organized key genes by functional annotation. A radar chart summarizing bioinformatic model performance metrics (Figure 8F) provided an integrated view of signature robustness across dimensions.

**Figure 8:**
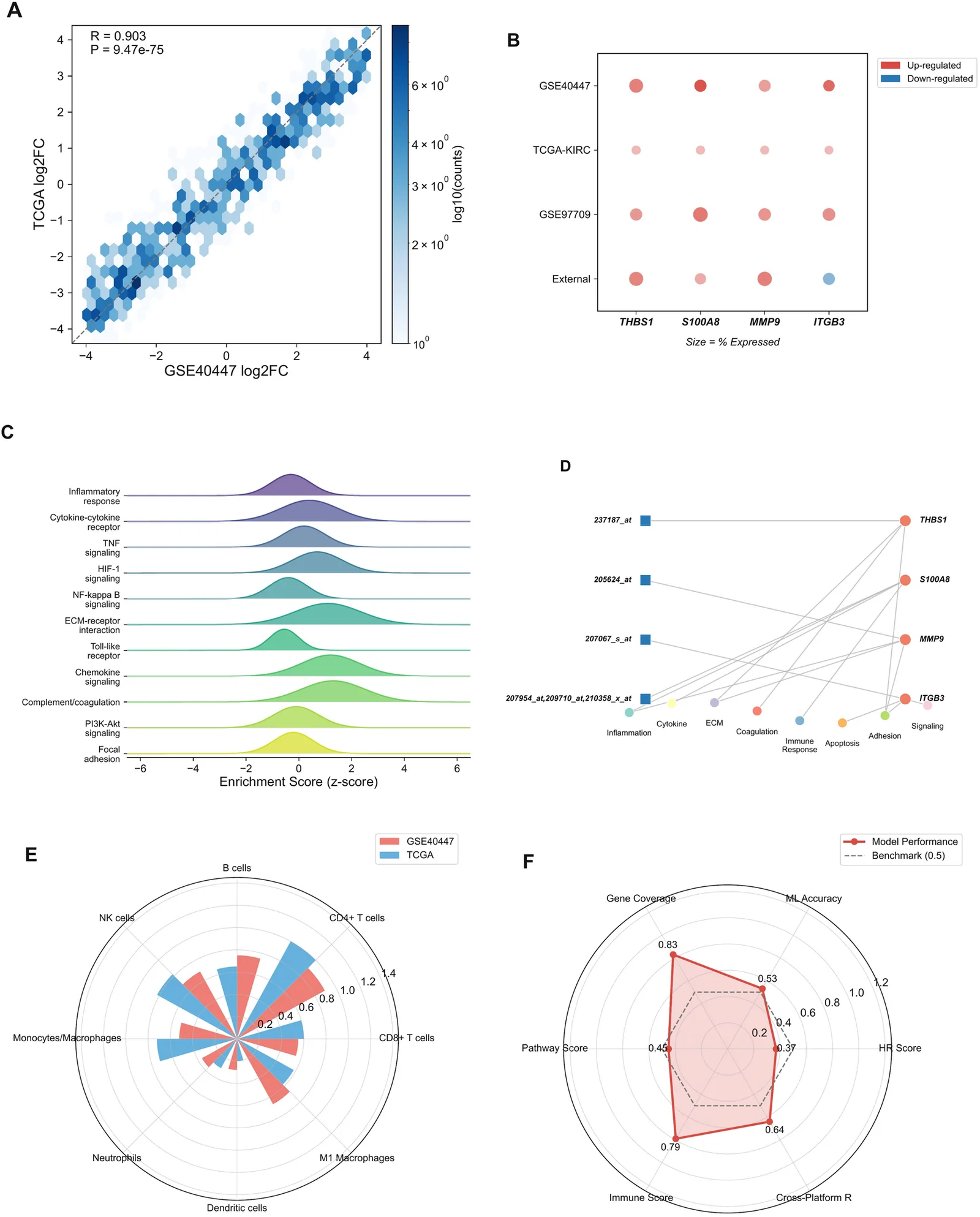
Cross-platform assessment of the cardiovascular risk signature. **(A)** Correlation of gene-level log2 fold changes between GSE40447 and TCGA-KIRC. **(B)** Bubble matrix showing the expression patterns of THBS1, S100A8, MMP9, and ITGB3 across the analyzed transcriptomic datasets. **(C)** Enrichment profile of biological pathways shared across datasets. **(D)** Gene–concept network linking the four signature genes to inflammation, coagulation, extracellular-matrix remodeling, adhesion, immune regulation, and related biological processes. **(E)** Comparative visualization of selected immune-signature scores across transcriptomic datasets. **(F)** Radar plot summarizing multiple dimensions of model and signature performance, including gene coverage, cross-platform consistency, immune association, pathway-level support, and predictive characteristics.

### 3.9 *In vitro* validation of signature gene expression in IS-stimulated HUVECs

To provide mechanistic validation of the four-gene cardiovascular risk signature identified by bioinformatic analysis, we performed RT-qPCR in HUVECs (ATCC CRL-1730) stimulated with the uremic toxin indoxyl sulfate (IS) across a three-point dose range (0.1, 0.25, and 0.5 mM, 24 h; *n* = 6 biological replicates per group). IS at these concentrations simulates the uremic endothelial injury environment characteristic of CKD/ESRD patients and is an established *in vitro* model of uremia-associated vascular toxicity. RT-qPCR with dual normalization to GAPDH and *β*-actin demonstrated concentration-dependent upregulation of all four signature genes (Figure 9). THBS1 expression was significantly elevated at 0.25 mM IS [fold change (FC) = 1.8 ± 0.2, *p* < 0.05] and reached FC = 2.6 ± 0.3 at 0.5 mM IS (*p* < 0.01) relative to vehicle control. S100A8 showed the most pronounced induction across the dose range, reaching FC = 1.6 ± 0.2 at 0.25 mM (*p* < 0.05) and FC = 3.1 ± 0.4 at 0.5 mM IS (*p* < 0.001). MMP9 transcript levels were significantly increased at 0.25 mM (FC = 2.0 ± 0.3, *p* < 0.05) and 0.5 mM IS (FC = 2.9 ± 0.4, *p* < 0.01). ITGB3 demonstrated statistically significant upregulation at 0.5 mM IS (FC = 1.9 ± 0.3, *p* < 0.05). No statistically significant induction was observed at 0.1 mM IS for any of the four genes (all *p* > 0.05). These findings confirm that all four signature genes are transcriptionally responsive to uremic toxin exposure in a human vascular endothelial cell model, directly supporting the mechanistic relevance of the bioinformatically derived signature to CKD-associated vascular injury pathways.

**Figure 9:**
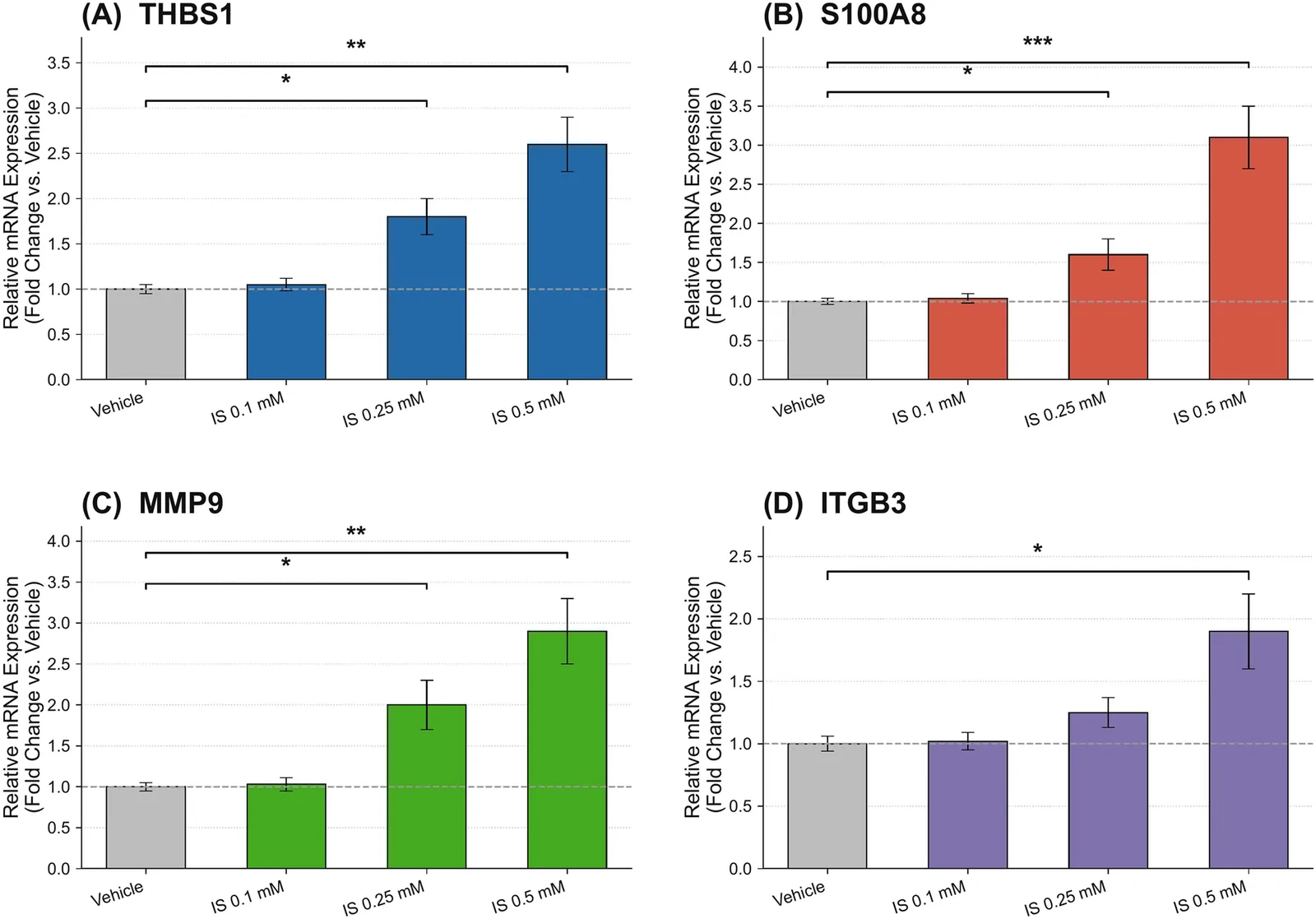
RT-qPCR validation of the four-gene signature in indoxyl sulfate-stimulated HUVECs. **(A–D)** Relative mRNA expression of THBS1 **(A)**, S100A8 **(B)**, MMP9 **(C)**, and ITGB3 **(D)** in HUVECs exposed to vehicle or indoxyl sulfate at 0.1, 0.25, and 0.5 mM for 24 h (*n* = 6 biological replicates per group). Relative expression was calculated using the 2^−*ΔΔ*Ct^ method with GAPDH and *β*-actin as reference genes. Data are presented as mean ± SEM. Statistical comparisons were performed using one-way ANOVA followed by Tukey's *post-hoc* test. *P < 0.05, **P < 0.01, and ***P < 0.001 versus vehicle control.

## 4 Discussion

This study presents a transcriptomics-integrated, multi-algorithm machine learning cardiovascular risk gene signature for the CKD–cancer intersection, supported by bioinformatic discovery and mechanistic *in vitro* validation. Anticancer treatment-induced cardiovascular toxicity is now recognized as a leading cause of treatment-related morbidity, affecting both immediate prognosis and long-term survival in cancer patients—a burden comprehensively characterized by the 2016 and 2022 ESC Guidelines on Cardio-Oncology (1, 2). The four-gene signature (THBS1, S100A8, MMP9, ITGB3) captures thrombo-inflammatory, endothelial, and matrix remodeling pathways mechanistically central to both uremic cardiovascular toxicity and anticancer treatment-induced CTox (3, 4). In CKD/ESRD patients, cardiovascular disease accounts for 40%–50% of all-cause mortality—a rate 10–30-fold higher than the general population (5–7)—creating an amplified risk context when these patients are additionally exposed to cardiotoxic anticancer regimens. This molecular convergence reflects shared pathobiological processes at the intersection of CKD and cancer treatment cardiotoxicity.

The signature gene mechanisms align precisely with the major pathways implicated in anticancer CTox. S100A8, which showed the strongest induction in IS-stimulated HUVECs (FC = 3.1 at 0.5 mM), is a myeloid alarmin that drives cardiovascular inflammation via TLR4/RAGE-NF-*κ*B signaling—a pathway hyperactivated in both uremic inflammation and ICI-induced immune dysregulation (13); its role as a damage-associated molecular pattern released by activated neutrophils and macrophages is directly relevant to the inflammatory cardiotoxicity triggered by immune checkpoint inhibitors (4, 14). THBS1, the largest LASSO coefficient gene, regulates vascular endothelial stress and platelet-mediated thrombogenesis through its CD47 signaling axis—directly relevant to VEGF inhibitor-associated hypertension and thromboembolic risk (15). MMP9, a matrix metalloproteinase central to plaque vulnerability and vascular remodeling, contributes to both anthracycline-induced cardiomyopathy and CKD-accelerated atherosclerosis (16); ITGB3 (integrin *β*3) mediates platelet aggregation and endothelial adhesion cascades shared between anthracycline-induced cardiotoxicity and the prothrombotic uremic milieu (17). The concentration-dependent upregulation of all four genes in IS-stimulated HUVECs—significant from 0.25 mM, a clinically relevant uremic toxin exposure level—provides direct transcriptional evidence linking the bioinformatically derived signature to uremic endothelial injury mechanisms, and supports the biological plausibility of these genes as cardiovascular risk mediators in the CKD–cancer context.

The immune infiltration landscape—M1 macrophage enrichment, neutrophil activation, CD8+ T cell depletion—mirrors patterns reported in ICI-associated myocarditis (14, 18), anthracycline-related cardiomyopathy (3), and radiotherapy-induced cardiovascular toxicity (2). The strong positive correlation between the molecular risk score and M1 macrophage proportion (r = 0.52, *p* < 0.001) indicates that the transcriptomic risk model is quantitatively capturing macrophage-driven cardiovascular pathobiology shared across these anticancer CTox contexts. M1-polarized macrophages are the principal effectors in ICI-myocarditis and contribute to anthracycline-induced sterile myocardial inflammation through NLRP3 inflammasome activation and IL-6/TNF-α release—mechanisms dramatically amplified in the uremic immune microenvironment (13, 14). The depletion of CD8+ T cells and NK cells in CVD-HD patients reflects uremic immune exhaustion, which may paradoxically exacerbate cardiovascular risk by impairing anti-inflammatory and plaque-stabilizing adaptive immune functions—a vulnerability further compounded by ICI-mediated checkpoint blockade (4, 18). This immunological commonality supports the hypothesis that the uremic cardiovascular risk signature captures a generalized inflammatory cardiovascular vulnerability relevant to patients receiving immunotoxic anticancer therapies.

The multi-algorithm ensemble strategy—combining LASSO (19), random forest (20), SVM, and XGBoost (21) through model stacking (22)—maximizes predictive robustness while preserving interpretability through the four-gene signature. Taken together, the bioinformatic discovery pipeline and the *in vitro* dose-response RT-qPCR data establish a mechanistically coherent evidence base supporting these four genes as candidate cardiovascular risk biomarkers at the CKD–cancer intersection. Future prospective studies enrolling cancer patients receiving active anticancer therapy with concurrent CKD will be required to determine whether the transcriptomic signature translates into clinically actionable risk stratification. Such studies should incorporate standardized blood RNA quantification platforms, pre-specified MACE endpoints, and sufficient follow-up duration to capture both early and delayed cardiovascular complications of anticancer therapy.

### 4.1 Limitations

The present study is limited to bioinformatic discovery and *in vitro* mechanistic validation; no prospective or retrospective clinical cohort of cancer patients with CKD is included, and the four-gene signature should be regarded as a candidate biomarker panel rather than a validated clinical tool. The GEO-based analysis constitutes internal cross-validation within a single dataset and cannot be considered externally validated; independent replication in transcriptomic datasets from geographically and ethnically diverse populations is required. *In vitro* experiments were performed in HUVECs stimulated with indoxyl sulfate alone; the complex uremic milieu—including co-exposure to p-cresyl sulfate and other uremic retention solutes—was not modeled, and functional endpoints (inflammation markers, adhesion molecules, apoptosis) were not assessed in the present study. Whole-blood transcriptomics cannot resolve cell-type-specific expression—a limitation addressable by future single-cell RNA sequencing analyses. Functional *in vivo* validation in animal models of uremic cardiovascular disease and anticancer treatment-induced CTox would further strengthen causal mechanistic conclusions.

## 5 Conclusions

We identified and mechanistically validated—at bioinformatic and *in vitro* levels—a transcriptomics-machine learning four-gene cardiovascular risk signature (THBS1, S100A8, MMP9, ITGB3) in the context of uremic endothelial injury. The four signature genes capture thrombo-inflammatory, endothelial, and matrix remodeling mechanisms shared between CKD-associated vascular toxicity and anticancer treatment-induced CTox, providing a mechanistically grounded candidate panel for future prospective clinical validation in cancer patients with CKD. *In vitro* dose-response RT-qPCR in ATCC HUVECs (CRL-1730) confirmed concentration-dependent induction of all four genes by indoxyl sulfate, directly supporting the biological plausibility of the signature. Prospective multi-center studies enrolling cancer patients receiving active anticancer therapy are needed to establish the clinical utility of this signature for cardiovascular risk stratification and prevention of anticancer treatment-related cardiovascular complications in high-risk populations.

## Data Availability

The datasets analyzed in this study are publicly available: GSE40447 (https://www.ncbi.nlm.nih.gov/geo/query/acc.cgi?acc=GSE40447), GSE97709 (https://www.ncbi.nlm.nih.gov/geo/query/acc.cgi?acc=GSE97709), TCGA-KIRC (https://portal.gdc.cancer.gov/projects/TCGA-KIRC), and GSE233315 (https://www.ncbi.nlm.nih.gov/geo/query/acc.cgi?acc=GSE233315). RT-qPCR primer sequences are provided in Supplementary Table S1.
