# Supplementary material for: Transcriptomics- and machine learning-based identification and mechanistic in vitro validation of a cardiovascular risk gene signature at the CKD–cancer intersection: a multi-omics approach
Source: Front Cardiovasc Med. 2026 Sep 14;13:1915820. doi: 10.3389/fcvm.2026.1915820 (PMC13616729; doi:10.3389/fcvm.2026.1915820)
Supplement: Supplementary file 1 [file Supplementaryfile1.docx]

**Supplementary Table S1. RT-qPCR primer sequences**

*Primer sequences used for IS-stimulated HUVEC experiments*

| **Gene** | **Forward primer (5′–3′)** | **Reverse primer (5′–3′)** | **Public source / accession** | **Role** |
| --- | --- | --- | --- | --- |
| THBS1 | TGCTATCACAACGGAGTTCAGT | GCAGGACACCTTTTTGCAGATG | PrimerBank 40317625c3; NM_003246 | Target |
| S100A8 | ATGCCGTCTACAGGGATGACCT | AGAATGAGGAACTCCTGGAAGTTA | OriGene HP206570; NM_002964 | Target |
| MMP9 | GGGACGCAGACATCGTCATC | TCGTCATCGTCGAAATGGGC | PrimerBank 74272286c3; NM_004994 | Target |
| ITGB3 | AGTAACCTGCGGATTGGCTTC | GTCACCTGGTCAGTTAGCGT | PrimerBank 47078291c3; NM_000212 | Target |
| GAPDH | ACAACTTTGGTATCGTGGAAGG | GCCATCACGCCACAGTTTC | Published HUVEC reference-gene study; NM_001256799 | Reference |
| β-actin (ACTB) | CGACAGGATGCAGAAGGAG | ACATCTGCTGGAAGGTGGA | Published HUVEC reference-gene study; NM_001101.3 | Reference |

**Note.** The primer sequences shown are the actual sequences used for RT-qPCR analysis and were selected from public qPCR resources/literature, with the corresponding accession information provided. Primer specificity, amplicon size/efficiency, and melt-curve specificity should be confirmed under the experimental HUVEC/indoxyl-sulfate conditions.

**Normalization.** GAPDH and β-actin were used as dual reference genes for normalization of the RT-qPCR data.
